# A practical method for mass quantification of microplastics in soil media using pyrolysis gas chromatography-mass spectrometry

**DOI:** 10.1016/j.mex.2025.103711

**Published:** 2025-11-05

**Authors:** Elham Faraji, Patricia Cabedo-Sanz, Ajit K. Sarmah

**Affiliations:** Department of Civil and Environmental Engineering, The Faculty of Engineering, The University of Auckland, New Zealand

**Keywords:** Py-GC/MS, FTIR, Microplastic, Mass quantification, Microplastic extraction, soil media

## Abstract

Microplastic (MP) contamination in soil media presents growing analytical challenges. We introduce a validated method for MP mass quantification using pyrolysis–gas chromatography/mass spectrometry (Py-GC/MS), targeting polyethylene (PE), polypropylene (PP), and polystyrene (PS) in synthetic and environmental soils. The method achieved low limits of detection (0.02–0.44 µg), strong linearity (R² > 0.995), and high recovery rates—86.1% (sandy), 90.7 % (loamy), and 99.6 % (sandy-loam). Cryomilling improved sample homogeneity and quantification accuracy (+3.2 %). Fourier transform infrared spectroscopy (FTIR) was used to confirm polymer identity with >85% match. The protocol was successfully applied to environmental samples from urban and agricultural soils in Auckland, New Zealand, demonstrating its robustness and field applicability. This practical workflow offers a reproducible, high-sensitivity approach suitable for routine microplastics monitoring across diverse soil matrices.

Py-GC/MS method achieved over 90 % accuracy for microplastic quantification.

Extraction protocols demonstrated recovery efficiencies of up to 99.6 %

FTIR complemented Py-GC/MS, confirming polymer identification with >85 % accuracy.

## Graphical abstract

**Figure:**
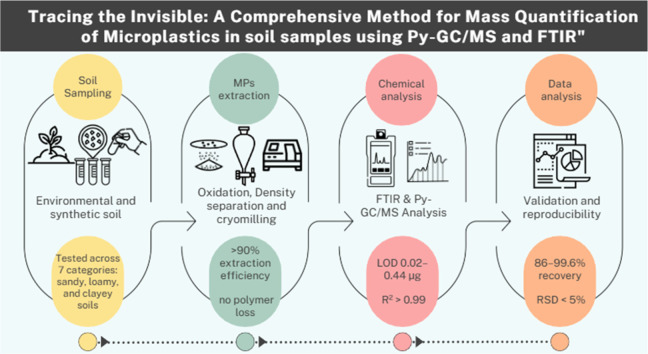

Specifications table

**Table:** 

Subject area	Environmental Science
**More specific subject area**	Microplastic quantification and polymer identification in soil environments
**Name of your method**	Quantitative analysis of microplastics in soil media using Pyrolysis-GC/MS with FTIR validation
**Name and reference of original method**	None.
**Resource availability**	None.

## Background

Microplastics (MPs) are increasingly recognized as emerging environmental contaminants with widespread presence in terrestrial ecosystems, including urban, agricultural, and stormwater-impacted soils. While the occurrence of MPs in aquatic systems has been extensively studied, the investigation of soil-based environments has lagged due to analytical challenges related to heterogeneous soil properties, presence of organic matter, and difficulties in isolating and quantifying MPs accurately. Given the rising concern about MPs entering food chains through soil-plant interactions, there is an urgent need for reproducible and validated analytical methods tailored to solid, complex environmental media.

Although several analytical techniques have been developed, including visual microscopy, FTIR (Fourier-transform infrared spectroscopy), and Raman spectroscopy, most are either qualitative or limited in resolution and sensitivity, especially when dealing with small particle sizes and complex soil matrices. Spectroscopy methods often require laborious particle isolation and pre-treatment and can be biased by matrix interference. Recent studies have emphasized that while spectroscopic approaches such as FTIR and Raman remain useful for particle identification, they often provide only count-based or semi-quantitative information, with strong dependence on particle size, shape, and polymer density [1,2]. Moreover, these techniques require extensive pre-cleaning and manual handling, which can result in contamination or loss of fine particles, particularly in soils rich in organic matter [3]. These limitations necessitate a more robust and quantitative technique to measure the mass concentration of polymer types in environmental soils. Py-GC/MS offers a promising alternative by enabling polymer-specific, mass-based quantification of microplastics through the identification of unique pyrolysis marker compounds [[4], [5], [6]].

Pyrolysis gas chromatography-mass spectrometry (Py-GC/MS) offers a promising alternative by enabling accurate mass-based quantification of polymers through their characteristic thermal degradation products. However, despite its application in a range of environmental samples, a complete, practical, and reproducible workflow for its use in diverse soil types remains poorly documented. In particular, the detailed procedural steps, challenges related to soil texture and composition, and polymer-specific recovery performance across various matrices have not been fully addressed in the existing literature.

The aim of this study was to develop, validate, and document a comprehensive and replicable method for the identification and quantification of common polymer types—polyethylene (PE), polypropylene (PP), and polystyrene (PS)—in soil environments using Py-GC/MS combined with FTIR. The method covers all procedural steps, including sample preparation, cryomilling for homogenization, density separation, addition of matrix modifiers (e.g., CaCO₃), pyrolysis conditions, and validation using both synthetic spiked soils and real environmental samples.

To test applicability across different soil textures and compositions, five synthetic soil media (sandy, loamy, sandy-loam, loamy-sand, and a raingarden soil mix) were spiked with reference MPs, and the method’s performance was validated via recovery efficiency, detection limit, and quantification accuracy. In addition, soil samples were collected from urban and agricultural sites in Auckland, New Zealand, to evaluate field applicability. FTIR was used as a supporting method for confirming polymer identity in both synthetic and environmental samples.

This article presents a step-by-step method with practical implementation in mind, suitable for academic researchers, environmental practitioners, and laboratories tasked with soil quality monitoring. The protocol demonstrated excellent linearity (R² > 0.995), low LOD (0.02–0.44 µg), and high recovery rates (up to 99.6 %). The method fills a critical gap by offering a detailed, reproducible, and field-validated procedure for MP mass quantification in soils, supporting regulatory and research efforts to track plastic pollution in terrestrial environments.

## Method details

### Materials

A commercially prepared standard blend of the 12 most frequently used plastic polymers was employed for the study (Frontier Laboratories Ltd., Japan). The mixture included polystyrene (PS), polyethylene (PE), polypropylene (PP), polyvinyl chloride (PVC), polyethylene terephthalate (PET), polycarbonate (PC), polyurethane (PUR), Nylon 6 (N-6), Nylon 66 (N-66), polymethyl methacrylate (PMMA), styrene-butadiene copolymer (SBR), and acrylonitrile butadiene styrene (ABS). The blend was prepared with 2 mg of calcium carbonate (CaCO_3_) as a non-reactive diluent. The precise amounts of each polymer present in the blend are listed in supplementary material (Table SM1).

Polyethylene, polystyrene, and polypropylene were selected for analysis due to their widespread use, low density, and high prevalence in both aquatic and terrestrial environments. Their large surface area and hydrophobicity enable them to adsorb and concentrate harmful substances such as heavy metals and organic pollutants, posing significant ecological risks. In the absence of certified standards, fresh polymers—LLDPE, HPP, and HIPS—sourced from Avient Ltd. (Auckland, New Zealand), ranging from 500 µm to 1 mm in size, were used as reference materials. Further details are provided in Supplementary material (Table SM2).

### Synthetic soil-based medium

Various synthetic soil-based medium was prepared to simulate different environmental conditions for microplastic quantification and to assess MPs extraction efficiency across diverse soil media mixtures. All medium were sourced from a registered and approved supplier based in Auckland, New Zealand. A total of 5 synthetic soil media mixtures were used as shown in Figure SM2. These included sandy soil media (80 % sand, 10 % Waikato topsoil media, and 10 % pumice), loamy soil media (40 % sand, 30 % Waikato topsoil media, 20 % compost, and 10 % pumice), sandy loam (60 % sand, 20 % Waikato topsoil media, 10 % compost, and 10 % pumice), and clay loam (30 % sand, 40 % Waikato topsoil media, 20 % compost, and 10 % gravel). Each soil media composition was designed to reflect varying levels of drainage, water retention, and aeration. These mixtures, along with a rain garden mix, provided a controlled setting to assess the efficiency of microplastic extraction and quantification across different soil media types. All soil media samples were spiked with known quantities of LLDPE, HPP, and HIPS microplastics sourced from Avient (New Zealand), ensuring a consistent concentration of microplastics for recovery and analysis. Each soil media was also tested as a blank sample using FTIR and Py-GC/MS to confirm that no microplastics were present in the original sources.

### Environmental soil samples

**Fig. 1:**
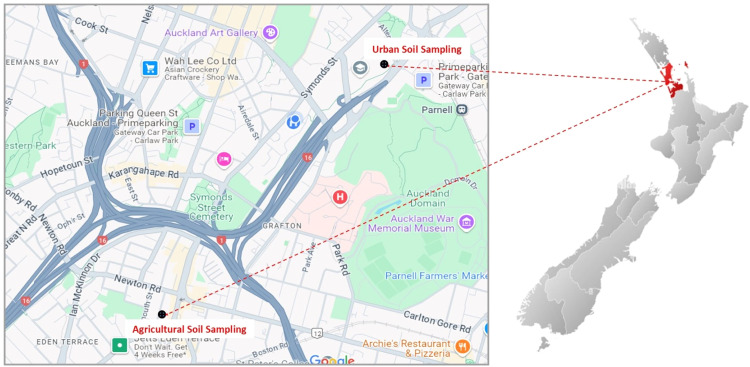
Environmental soil sampling in central Auckland, New Zealand.

Soil samples were collected from a depth of 10 cm at four separate locations within central Auckland, as depicted in Fig. 1, to test and implement a developed method for identifying and measuring microplastics in soil. Three of the sites were urban areas prone to contamination from urban runoff and traffic pollutants: a parking area near the roadside, a curb positioned above a stormwater catchpit, and soil near a pipe outlet. The fourth location, an urban farm, provided a different setting with no exposure to direct urban runoff. These varied sites were selected to evaluate the method's ability to identify and quantify microplastics under different environmental conditions and land uses.

### Extraction and pretreatment of MPs

**Fig. 2:**
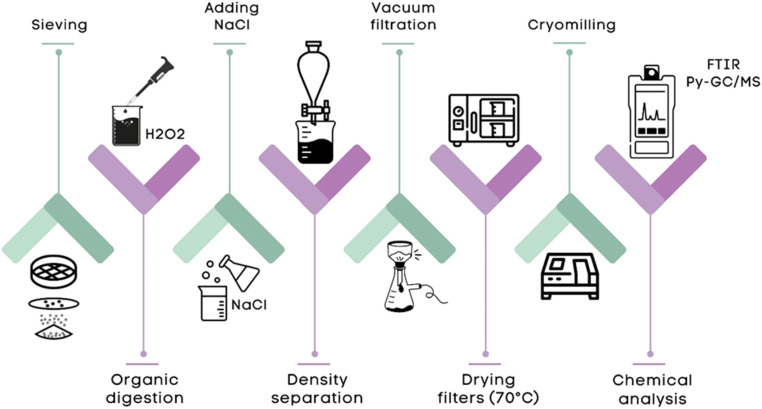
MPs digestion and extraction from soil media samples.

To extract MPs from soil media samples, a standardized method was employed with some modifications for each sample type and a schematic diagram is shown in Fig 2. For soil media samples, sieving was performed initially to remove larger particles and ensure uniformity in grain size. Following this, both the sieved soil media and water samples underwent organic digestion using hydrogen peroxide (H₂O₂), to effectively break down the organic matter to minimize interference in subsequent analysis.

A density separation was performed using a sodium chloride (NaCl) solution, prepared by dissolving 365 *g* of NaCl in 1 Liter of Milli-Q water. The solution was stirred on a magnetic stirrer for at least 2 h under a fume hood to ensure complete dissolution. After that, the solution was filtered to eliminate any extraneous particles that might interfere with the microplastics analysis. This method is consistent with the protocols used in recent studies that evaluated the efficiency of NaCl solutions for separating microplastics from sediment [3]. This density-based separation allowed the microplastics, which are less dense, to float, while the denser particles, such as soil media components, settled at the bottom. This approach was particularly effective for soil media sample processing. This procedure was repeated three times to enhance recovery. Vacuum filtration was applied to the soil media phase after density separation to capture microplastics on the filter (Glass Fiber Membrane Filter, 0.7 μm Pore Size).

Following filtration, the filters were dried at 70 °C to ensure no residual moisture was left that would otherwise affect the final analysis. The last step was cryomilling / freezer milling, which is a crucial step in the extraction process, ensuring the complete homogenization of microplastics both extracted and remaining within the soil media matrix. The procedure was performed using a Total Lab Cole-Parmer instrument, set to 9, for three cycles, each lasting three minutes. This process is essential for reducing the particle size, thereby enhancing the precision of the subsequent analytical stages. Additionally, the inclusion of CaCO₃ (2–2.2 mg) during milling contributes to a uniform sample mixture, which is critical for accurate analysis. This step not only improves sample Homogeneity but also significantly minimizes variability, leading to more reliable data on microplastic quantification. Finally, FTIR and Pyrolysis-GC/MS were used as the final step to precisely identify and quantify microplastics in both soil media and water samples, providing robust analytical results across different environmental matrices.

### Method validation using FTIR

The FTIR analysis employed Attenuated Total Reflectance-Fourier Transform Infrared Spectroscopy (ATR-FTIR) using a Bruker Tensor 37 spectrometer equipped with a DLaTGS (deuterated l-alanine doped triglycine sulphate) detector and a diamond ATR crystal, enabling direct and efficient analysis of soil media samples. Microscopy techniques, including FTIR microscopy with a Hyperion 3000 microscope, further enhanced detection sensitivity. Spectra were recorded between 4000 and 600 cm⁻¹ with 4 cm⁻¹ resolution, and data analysis was performed using OPUS 8.5.29 software, supported by reference libraries. This streamlined ATR-FTIR method accelerated the sample preparation process while ensuring accurate identification of microplastics in environmental samples. Full operating conditions are detailed in supplementary information (Table SM3). The use of ATR-FTIR, with a concave component is designed to hold powdered samples, enhances the accuracy of the spectra, and ensures thorough assessment of all solid or powder samples (Figure SM3).

### Method validation using Pyrolysis-GC/MS

Microplastics analysis was performed using Pyrolysis-GC/MS, utilizing a Shimadzu GCMS-QP2020NX gas chromatograph / mass spectrometer. The system was equipped with an EGA/PY-3030D Frontier Lab pyrolyzer, set to a pyrolysis temperature of 600 °C for a duration of 0.2 min, and an interface temperature of 300 °C. The GC inlet was set at 300 C° and operated in split mode (50:1), with helium as the carrier gas at a flow rate of 1.04 mL/min, using an Ultra Alloy Capillary Column (30 m x 0.25 mm ID x 0.25 µm film thickness; Frontier Labs Ltd.). The oven temperature program began at 40 °C, held for 2 min, followed by a ramp of 20 °C/min to 320 °C, sustained for 10 min, with a backflush system which was activated at 20 min and until the end of the run to remove contamination at the column head and avoid ghost peaks in subsequent runs. The mass spectrometer was operated with electron ionization at 70 eV, scanning in the range of *m/z* 29–400 at a speed of 2000, with interface and ion source temperatures of 320 °C and 230 °C, respectively. This configuration allowed the identification and quantification of microplastics across multiple soil media types, ensuring robustness in analysis. Detailed Py-GC/MS conditions are summarised in the supplementary information (Table SM4). Polymer-specific pyrolysis markers were identified and verified based on established literature [4,5] and experimental calibration using the FrontierLab F-Search Microplastics database. Diagnostic fragments used for quantification included *m/z* 83 (1-dodecene) for polyethylene (PE), *m/z* 126 (2,4-dimethyl-1-heptene) for polypropylene (PP), and *m/z* 91 (styrene trimer) for polystyrene (PS). These marker compounds and their retention times are summarized in Table SM5. Blank analyses were performed for each soil type (*n* = 15 total), following identical pyrolysis conditions. No characteristic polymer peaks were detected within the corresponding retention-time windows, confirming that co-pyrolysis or matrix-derived interferences were negligible. The average blank signal intensity for all markers remained < 0.5 % of the lowest calibration standard, indicating high analytical specificity [7]. Although the Py-GC/MS quantification principle is established, the novelty of this study lies in its integrated analytical configuration that combines cryogenic milling for reproducible sample homogenization, CaCO₃ as an inert diluent to suppress co-pyrolysis artifacts, and FTIR confirmation for polymer identity. This optimized workflow achieved detection limits of 0.02–0.44 µg and recovery efficiencies of 86–99.6 %, outperforming previous studies [1,6].

The Py-GC/MS method was rigorously validated to ensure precision, accuracy, and sensitivity for microplastic quantification. Repeatability was determined by analyzing five replicates of 0.4 mg and 4 mg microplastic standards (MPs-CaCO₃), representing the minimum and average masses used for calibration, in a single day using the same instrument. Reproducibility was assessed through triplicate analyses of these standards over three consecutive days to evaluate the inter-day precision.

Calibration curves for each polymer demonstrated strong linearity, with coefficients of determination (R²) being consistently above 95 %, confirming the reliability of the calibration across the full concentration range (Fig. SM3). Limits of Detection (LOD) and Limits of Quantification (LOQ) were calculated using the standard deviation of the lowest measured concentration and the slope of the calibration curve, according to the following equations:

• LOD = 3.3 × (σ/S)
• LOQ = 10 × (σ/S)

where *σ* represents the standard deviation of the response and *S* denotes the slope of the calibration curve. The LOD indicates the lowest concentration at which microplastics can be reliably detected, while the LOQ defines the minimum level at which they can be accurately quantified. Values below the LOQ may still be detected but cannot be measured with certainty, which could affect data interpretation in environmental monitoring.

The accuracy of the method was further evaluated by comparing measured concentrations of synthetic samples against known concentrations. The percentage accuracy was calculated as:

• Accuracy ( %) = [(Measured Value−True Value​)/True Value] × 100

The accuracy varied across the polymers studied with most showing deviations within 10 %, indicating robust performance. Accuracy can also be applied to adjust the mass quantification of microplastics, ensuring that the reported results from the final assessment are corrected for any discrepancies identified during the validation process.

### Pyrolysis-GC/MS calibration and pyrolyzate identification

To ensure the reliability of the method, a calibration curve was established using five different concentrations of 0.4, 1, 2, 4 and 8 mg of standard mixture of MPs and CaCO_3_. For robust linearity assessment, each concentration should be measured at least three times to mitigate intra-assay variability, as this process enables precise calculation of the average response and its standard deviation at each concentration.

To enhance the robustness of our microplastic analysis, an extensive literature review was initially conducted to create a comprehensive database of distinctive pyrolysis products and their corresponding indicator ions for twelve plastic polymers [4,5]. This carefully curated database was instrumental in analysing a standard mixture containing 8 mg of MPs-CaCO_3_ under controlled Py-GC/MS conditions, and the pyrograms are presented in Figure SM1. For the analysis, we utilized GCMS Solutions and F-search software, which facilitated the comparison of generated pyrograms with our in-house database and validated them against standard libraries from Frontier Lab [[8], [9]]. This comparative analysis allowed for the precise identification of specific and abundant compounds characteristic of each polymer, as detailed in Table SM5, thereby significantly enhancing the accuracy of our identification and quantification processes. The integration of these advanced software tools underscores the reliability of our methodological approach in polymer identification, leveraging both tailored in-house and widely recognized external databases.

### Quality assurance and control

To maintain the integrity of our analyses and minimize plastic contamination, a comprehensive set of Quality Assurance and Control (QA/QC) measures were meticulously applied throughout the entire process. Sampling procedures were executed by operators wearing cotton lab coats and gloves, featuring stainless steel surfaces and furniture. Pretreatment processes, encompassing extraction, purification, and filtration, were conducted under a decontaminated steel fume hood within this controlled environment. Thorough decontamination protocols were implemented for all steel ware, glassware, and equipment, ensuring rigorous standards were met. Filters, post-filtration, were stored in decontaminated glass Petri dishes under the fume hood and later transferred to the Micro-FTIR laboratory, covered with aluminium foil to prevent external contamination. Reagent and procedural blanks were tested for MPs contamination, running in triplicate, with no observed plastic particles on any of the blanks. These robust QA/QC procedures underscore our commitment to delivering reliable and accurate results while adhering to the highest laboratory standards.

## Method validation

### Extraction efficiency

**Fig. 3:**
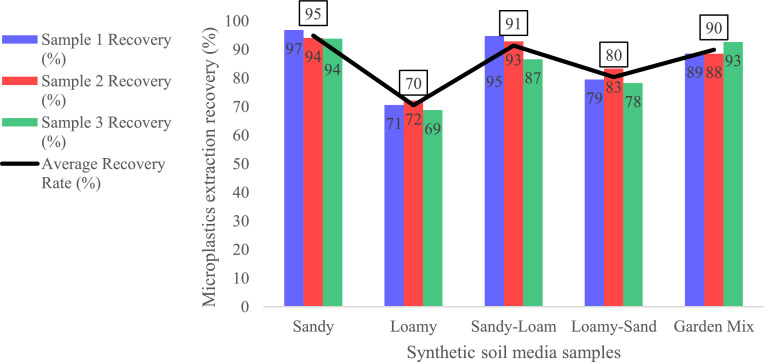
Recovery rates across different Synthetic Soil media samples.

Extracting microplastics from soil media is a complex process, with recovery rates differing greatly depending on the method and polymer type. Sodium chloride (NaCl) is one of the most commonly used solutions for density separation due to its affordability and safety. It performs well for light polymers like PE and PP, achieving recovery rates of 80–100 %, but is less effective for denser polymers such as PVC and PET, which respond better to NaI or ZnCl_2_ solutions [3,10,2]. Sodium bromide has also shown high recovery rates of 85–100 % for various polymers [10]. The extraction efficiency of microplastics across various synthesized soil media types, as illustrated in Fig. 3, reveals distinct variability based on soil media composition. For example, sandy soil media exhibited the highest recovery rate, reaching 97 %, which can be attributed to its low organic matter content and coarse texture. These characteristics likely facilitated the separation and recovery of microplastics. In contrast, loamy soil medium demonstrated the lowest recovery rates, ranging from 69 % to 72 %. This reduction in recovery could be attributed to the finer particle size and increased organic content of loamy soil medium, which could result in microplastics binding more strongly to the soil media matrix, making their extraction more challenging.

Intermediate recovery rates were observed for sandy-loam and loamy-sand soil medium, with values ranging between 78 % and 95 %. Given these soil medium present a mix of sand and organic components; they still pose moderate extraction challenges. For instance, the garden mix soil media showed consistently high recovery rates, exceeding 90 %, indicating that well-optimized extraction protocols can effectively recover microplastics even in more complex, mixed soil media compositions. While overall recovery rates exceeded 80 % for most soil media types, the results suggest that further optimization of extraction protocols is necessary for soil medium with higher organic matter content to enhance microplastic recovery efficiency.

### Calibration curves for microplastics mass quantification

In validating the microplastic quantification method via Py-GC/MS, calibration curves for each polymer showed excellent linearity (R² ≈ 1), underscoring the method's accuracy in correlating polymer concentration with detector response (Figure SM4). For instance, the calibration curve for PP achieved R² value of 0.999, while the low LOD for PS at 0.05 µg highlights the method’s sensitivity in detecting trace MPs. These calibration boundaries are critical for reliable quantification; however, exceeding the highest concentration in the calibration curve risks detector over-saturation, which can compromise accuracy. Therefore, to mitigate this aspect, freeze milling of the sample is essential, as it ensures homogeneity in the sample and prevents overloading of the instrument, allowing representative measurements of MPs without error from concentration inconsistencies. Figure SM4 demonstrates this precision, with PS displaying a high recovery rate of nearly 99 %, while PE and PP showed reliable recoveries. Overall, the average recovery rate across samples was 95 %, reflecting the method’s reliability and accuracy.

### Method validation

The validation of the analytical method demonstrated strong performance across the 12 polymers tested, as shown in Table 1. The high R² values, consistently close to 1.0, confirm excellent linearity, a key factor in ensuring reliable and accurate results. The method showed particularly strong sensitivity for polymers such as PS and SBR, where low LOD and LOQ were recorded, highlighting the method's ability to detect even small amounts of these polymers. In contrast, polymers such as PET and N6 required higher concentrations for detection and quantification, indicating some variability in sensitivity based on polymer type.

**Table 1:** Validation parameters of the 12 target Plastic Polymers.

Polymer	Indicator ion (*m/z*)	R ^2^	LOD (μg)	LOQ (μg)	Repeatability ( %RSD)		Reproducibility ( %RSD)		Accuracy ( %)	
					0.4 mg	4.0 mg	0.4 mg	4.0 mg	0.4 mg	4.0 mg
PE	82	0.997	0.44	1.32	1.20	1.93	2.84	6.09	−9.09	−1.76
PP	126	0.999	0.28	0.84	0.96	1.21	3.28	5.95	−7.88	−1.46
PS	91	0.999	0.05	0.15	1.22	0.96	3.91	5.39	−22.36	−2.65
ABS	170	0.999	0.26	0.80	4.04	1.56	6.43	5.20	−9.57	−2.29
SBR	104	0.999	0.12	0.35	0.88	1.29	3.12	5.17	−19.11	−1.50
PMMA	100	0.998	0.02	0.07	2.09	1.44	4.72	5.15	−23.97	−2.14
PC	134	0.997	0.13	0.40	3.68	0.94	6.29	5.85	6.38	0.74
PVC	128	0.999	0.33	1.00	1.63	1.54	2.48	5.40	−19.77	−0.53
PU	198	0.993	0.04	0.14	8.05	6.50	8.95	10.92	−10.20	−10.80
PET	182	0.994	0.40	1.20	6.16	1.82	6.82	6.55	−0.54	−0.71
N6	113	0.999	0.07	0.22	3.61	0.54	5.79	5.38	−13.21	−1.43
N66	84	0.999	0.14	0.42	1.63	0.31	2.44	5.52	−16.59	−1.28

In terms of precision, repeatability and reproducibility were well-maintained across the majority of polymers, with low percent relative standard deviations ( %RSD) observed, particularly for PE and PP. This suggests a high degree of reliability and consistency in the method, even when testing was conducted across different days. However, some materials, such as PU and ABS, exhibited higher %RSD values, suggesting that further refinement of analytical method might be beneficial for these polymers to improve consistency. The robustness of the method was particularly evident with polymers like PC and PVC, which showed minimal variation, reinforcing the method’s reproducibility.

Accuracy of the results revealed that some polymers, including PS and PMMA, tended to be slightly underestimated when compared to calibration kit standards, as indicated by negative values of accuracy. This suggests that further calibration adjustments could enhance the method’s accuracy, especially for these materials. However, polymers like PC and PET exhibited accuracy levels that aligned closely with expected concentrations, supporting the method’s overall reliability.

### Application of method

#### FTIR analysis of synthetic soil-based media

**Fig. 4:**
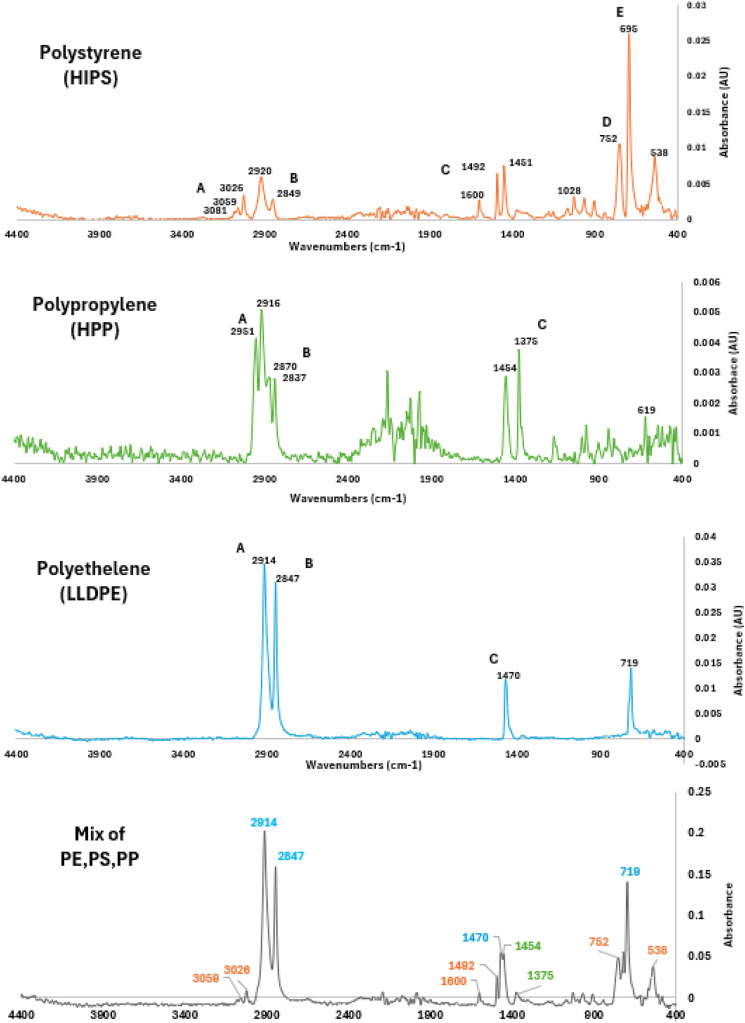
Wavelet of reference polymers of LLDPE, HPP and HIPS and mixture of them extracted from synthetic soil media sample.

The FTIR analysis of synthetic soil medium spiked with microplastics yielded distinct absorption peaks corresponding to the polymers' molecular structures (Fig. 4). Peaks such as 3056 cm⁻¹ for polystyrene (PS), 2914 cm⁻¹ for polyethylene (PE), and 2916 cm⁻¹ for polypropylene (PP) were consistently identified in both individual polymer spectra and mixed samples, demonstrating the method's efficacy. The application of ATR-FTIR, with a concave part to hold powdered samples, ensured excellent contact between the samples and the crystal, producing high-quality spectral data. Polymer identification was confirmed for correlations exceeding 80 % for extracted microplastics and 50 % for soil media samples containing microplastics. In the presence of soil media, wavelengths fluctuated, and absorbance values varied when compared to pure polymer spectra, reflecting the impact of the soil media matrix. Correlation scores from OPUS library searches, ranging from 49.3 % to 68.6 %, provided additional validation of successful polymer identification in soil media samples (Figure SM5).

**Fig. 5:**
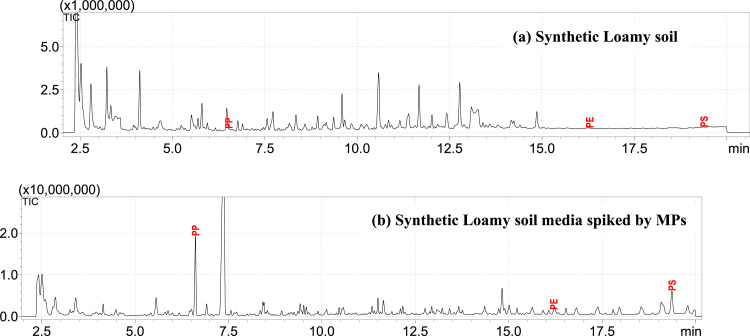
Pyrograms of Synthetic Loamy soil media (10 mg): (a) Without any spiked MPs, (b) With spiked LLDPE, HIPS and HPP. 4.0 mg of CaCO_3_ were added to each sample.

Despite the complexity of the soil media matrix, the analysis accurately identified the spiked microplastics in all samples, demonstrating above 50 % match with reference spectra (Figure SM6), confirming that the FTIR method is effective in overcoming soil media interferences, ensuring reliable microplastic identification. Furthermore, this non-destructive, fast technique is highly recommended for initial identification before proceeding with detailed quantification using Py-GC/MS, enhancing the overall workflow efficiency for soil media-based microplastic analysis. This finding is illustrated in Fig. 5 in following section showing clear identification of microplastics in synthetic soil medium.

#### Py-GC/MS analysis of synthetic soil-based media

The synthetic soil media samples, both with and without MPs, were analyzed using Py-GC/MS in the presence of CaCO₃, and the resulting pyrograms are presented in Fig. 5. As clearly shown, the synthetic loamy soil media detected PE, PP, and PS positively; however, their concentrations were below the Limit of Quantification (LOQ) but exceeded the Limit of Detection (LOD), indicating their negligible presence in the soil media sample. For polymer identification, all pyrolyzates listed in Table 1 were reviewed and confirmed before proceeding with quantification using the calibration curve. The detailed process for this verification, utilizing F-Search software, is outlined in the 'SM Polymer Approval' section in supplementary material (Fig SM7-SM9). The pyrogram of synthetic loamy soil media spiked with reference MPs demonstrated successful identification and quantification of all polymers with more than 90 % accuracy when compared to the spiked concentrations.

Table 2 presents the results of MP quantification in synthetic soil medium spiked with LLDPE, HPP, and HIPS using Py-GC/MS. In Synthetic soil media 2 (Loamy), recovery rates were 87 % for LLDPE, 105 % for HPP, and 81 % for HIPS, resulting in an average recovery of 90.7 %, indicating good accuracy across all polymers. However, Synthetic soil media 1 (Sandy) displayed lower recovery rates, particularly for LLDPE (56 %), with an overall average recovery of 86.1 %, suggesting that accurately quantifying microplastics in sandy soil medium may present more challenges. In contrast, Synthetic soil media 3 (Sandy-Loam) demonstrated the highest recovery rates, with 113 % for LLDPE and an overall average recovery of 99.6 %, suggesting that this mixed soil media type performs well for microplastic quantification. The raingarden mix media (Synthetic soil media 5) showed relatively consistent recovery rates across polymers (ranging from 89 % to 91 %), resulting in an average recovery of 86.8 %, which indicates moderate accuracy.

**Table 2:** Pyrolysis accuracy in MPs Quantification in synthetic soil media samples spiked by reference LLDPE, HPP and HIPS (10 mg of soil media samples).

Soil Type	Spiked Amount (µg)			Recovered Amount (µg)			Recovery Rate of each polymer ( %)			Average Recovery Rate ( %)
	LLDPE	HPP	HIPS	LLDPE	HPP	HIPS	LLDPE	HPP	HIPS	
Synthetic soil1_Sandy	20	52	142	11	60	124	56	115	87	86.1
Synthetic soil2_Loamy	68	98	63	59	102	51	87	105	81	90.7
Synthetic soil3_Sandy-Loam	80	220	110	90	206	101	113	94	92	99.6
Synthetic soil4_Loamy-Sand	140	270	134	126	275	119	90	102	89	93.4
Synthetic soil5_Raingarden mix	212	247	270	190	199	245	89	80	91	86.8

The results also suggest that soil media texture plays a crucial role in influencing recovery rates. Sandy soil media (Synthetic soil media 1) exhibited the lowest accuracy, which could be attributed to its coarse texture and low organic content, reducing the retention of lighter MPs such as LLDPE during the extraction process. On the other hand, the mixed sandy-loam soil media (Synthetic soil media 3) achieved the highest accuracy, likely due to its intermediate particle size and moderate organic matter content, which enhanced retention of the microplastics. Loamy soil media (Synthetic soil media 2) also demonstrated strong performance, while the raingarden mix (Synthetic soil media 5) yielded consistent but slightly lower recovery rates, possibly due to its more complex composition. Among the tested media, sandy-loam soil achieved the highest extraction accuracy, while sandy soil initially showed the lowest, which improved significantly with cryomilling.

**Fig. 6:**
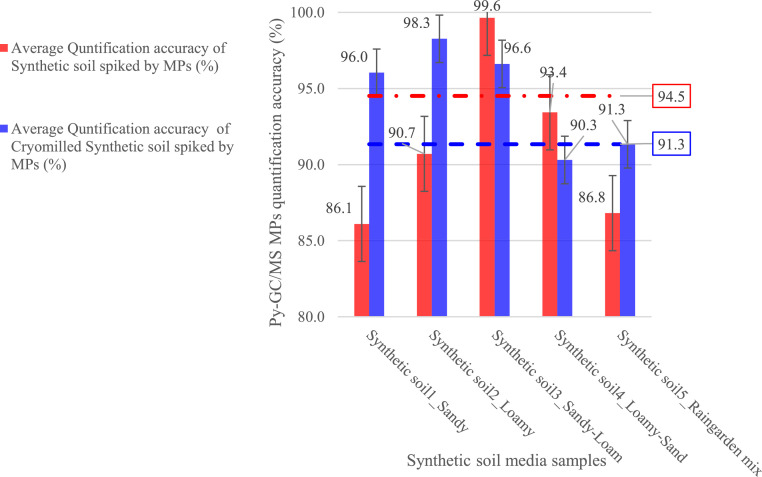
MPs mass quantification using Py-GC/MS Across Synthetic Soil Media Types.

Fig. 6 illustrates the mass quantification accuracy of microplastics (MPs) in synthetic soil media samples using the developed and validated Py-GC/MS method. Overall, the method demonstrated high accuracy, with an average efficiency of 91.3 % for non-cryomilled samples. Cryomilled samples showed enhanced performance, achieving an average accuracy of 94.5 %. Notably, in sandy soil media (Synthetic Soil 1), quantification accuracy increased from 86.1 % to 96 % following cryomilling. Similarly, in loamy soil media (Synthetic soil media 2), the quantification accuracy improved from 90.7 % to 98.3 %. For sandy loam soil media (Synthetic soil media 3), the accuracy rose from 93.4 % to 99.6 %, while in the raingarden mix (Synthetic soil media 5), it increased from 91.3 % to 93.4 %. The results highlight that cryomilling significantly enhances sample homogeneity, particularly in soil medium with mixed textures, by ensuring better distribution of MPs throughout the sample. The reduced error margins in cryomilled samples further indicate that this technique provides a more reliable and consistent quantification, reducing potential discrepancies caused by uneven microplastic distribution within the soil media matrix.

### Microplastics identification and quantification of environmental samples

Developed and validated extraction, identification and quantification methods were applied on soil environmental samples. The FTIR analysis of environmental soil samples (Figure SM11–12) provided valuable insights into the presence of specific microplastics in urban and agricultural settings. Peaks corresponding to PE and PP were distinctly identified in urban soils, such as pipe outlet and roadside parking areas. These findings align with the dominance of PE and PP in urban environments due to their widespread use in packaging and infrastructure materials. Additionally, PET was identified in curb and agricultural soils, suggesting contamination from textile fibers or plastic mulching. This quick, non-destructive method proves effective in providing evidence of microplastics in soil samples, even without prior extraction, making it a practical tool for initial identification in real-world environmental analyses.

The analysis presented in Table 3 demonstrates clear distinctions in MPs concentrations and types across various urban and agricultural soil samples analysed by Py-GC/MS using validated previously discussed method. The total ion chromatograms for the farm soil and roadside samples are presented in Figure SM13 and Figure SM14 respectively. Urban soils, particularly those near pipe outlets and roadside environments, exhibited the highest MP concentrations, with PP and PVC as the dominant polymers. In contrast, PS, ABS, PU, and N6 were absent across all samples, likely due to their lower prevalence or limited transport through stormwater systems, consistent with findings by reported recently [3,10]. Undetected MPs may be transported through stormwater pathways due to their physical properties or degrade during transit, preventing their deposition in urban soils.

**Table 3:** Mass of microplastics in environmental soil samples.

Polymer Type	Parking Roadside (µg/g)	Curb (µg/g)	Pipe Outlet Soil (µg/g)	Agriculture Soil (µg/g)
PE	1.2	0.72	0.62	0.29
PP	3.41	1.74	13.81	0.03
SBR	-	0.17	-	-
PMMA	0.45	3.01	-	-
PC	0.04	0.04	0.03	0.01
PVC	1.75	2	1.03	0.8
PET	0.34	0.36	0.5	0.14
N66	0.16	0.29	0.23	0.05

Pipe outlet soils showed a striking dominance of PP (13.81 µg/g), highlighting its role in urban pollution pathways. Roadside parking soils contained notable levels of PP (3.41 µg/g) and PVC (1.75 µg/g), along with smaller quantities of PE (1.2 µg/g), PMMA (0.45 µg/g), and PET (0.34 µg/g), reflecting diverse pollution sources, including packaging materials, textile fibres, and construction debris. Curb soils showed high levels of PMMA (3.01 µg/g) and PVC (2 µg/g), with trace amounts of SBR (0.17 µg/g) and PC (0.04 µg/g), indicating contamination from construction materials, rubber tires, and electronic components.

Agricultural soils exhibited significantly lower MP concentrations, with PE (0.29 µg/g) and PVC (0.8 µg/g) being the most prominent. These findings align with previous studies reported by [11,1], who found agricultural contamination was predominantly from plastic mulching and irrigation practices. The lower diversity and concentration of MPs in agricultural soils compared to urban samples emphasize the influence of land use and pollution sources.

The developed FTIR and Py-GC/MS methods proved effective for reliable MP identification and quantification, with sensitivity reflected in low LOD and LOQ, consistent with the findings reported recently [11]. Furthermore, this method was designed to comprehensively address all aspects of microplastic analysis, offering a detailed, step-by-step practical guide to ensure accuracy and reproducibility in both identification and quantification processes

## Limitations

### Interference mitigation and key learnings in microplastic analysis

Addressing the interferences in the microplastic quantification is paramount to ensure the accuracy and reliability of the results. The methodological refinements proposed include a focus on mitigating analytical variability—such as inconsistencies in pyrolysis temperature, and the flow rate of the carrier gas—and matrix effects from other organic materials that can obscure microplastic signals or create overlapping peaks. To combat these challenges, our measures include regular instrument calibration using standard reference materials and routine maintenance to ensure the consistent operation of the Py-GC/MS setup. As shown in Figure SM7, different parts of the pyrolyser unit and GC inlet get contaminated and would affect spectrum precision and quantification accuracy, underscoring the need of cleaning or replacing those parts after 100 runs. To address mass spectrometer detector drift, the MS was tuned regularly.

Practical lessons from our lab's experiences have been crucial in refining our approach. For instance, conducting a clean test measurement before each FTIR analysis ensures that the holder and crystal remain free from contamination, leading to more reliable and accurate results. We implemented a waiting period of two minutes after opening the Py-GC/MS injector to attach the sample cup prior to pyrolysis. This ensured that there was no residual air that could interfere with the tests and overload the MS detector. Regular cleaning of the equipment and bench areas, running blank checks before and after sample tests, and specific measures described earlier to handle samples containing high concentrations of MPs, helped to prevent false positives and contamination. This includes baking the column for 3 h at 330 °C if high concentrations are detected in blanks and using a freezer mill/ cryomill to ensure sample homogeneity.

Additionally, better results have been achieved by using CaCO_3_ as a diluent, which ensures more consistent sample homogeneity and minimizes interference. The meticulous calibration of the Py-GC/MS system has been enabled using a standard microplastics calibration kit, thereby enhancing the reliability and accuracy of the measurements. Such attention to detail during sample handling and analysis ensures that our findings contribute to valuable insights into the environmental impact of MPs, supporting our efforts to mitigate this pervasive issue.

## Ethics statements

This study does not involve human participants or animals. The author confirm that the work follows the ethical publication practices of MethodsX.

## Related research article

None.

## For a published article

None.

## CRediT authorship contribution statement

**Elham Faraji:** Conceptualization, Methodology, Software, Data curation, Writing – original draft. **Patricia Cabedo-Sanz:** Writing – review & editing. **Ajit K. Sarmah:** Supervision, Writing – review & editing.

## Declaration of competing interest

The authors declare that they have no known competing financial interests or personal relationships that could have appeared to influence the work reported in this paper.

## Data Availability

Data will be made available on request.

## Appendix Supplementary materials

Image, application 1

## References

[1] Li Z., Wang X., Liang S., Li H., Sun L. (2021). Pyr-GC-MS analysis of microplastics extracted from farmland soils. Int. J. Env. Anal. Chem..

[2] Radford F., Zapata-Restrepo L.M., Horton A.A., Hudson M.D., Shaw P.J., Williams I.D. (2021). Developing a systematic method for extraction of microplastics in soils [10.1039/D0AY02086A]. Anal. Methods.

[3] Duong T.T., Le P.T., Nguyen T.N.H., Hoang T.Q., Ngo H.M., Doan T.O., Le T.P.Q., Bui H.T., Bui M.H., Trinh V.T., Nguyen T.L., Da Le N., Vu T.M., Tran T.K.C., Ho T.C., Phuong N.N., Strady E. (2022). Selection of a density separation solution to study microplastics in tropical riverine sediment. Env. Monit. Assess..

[4] Fischer M., Scholz-Böttcher B.M. (2017). Simultaneous trace identification and quantification of common types of microplastics in environmental samples by pyrolysis-gas chromatography-mass spectrometry. Env. Sci. Technol..

[5] Santos L.H.M.L.M., Insa S., Arxé M., Buttiglieri G., Rodríguez-Mozaz S., Barceló D. (2023). Analysis of microplastics in the environment: identification and quantification of trace levels of common types of plastic polymers using pyrolysis-GC/MS. MethodsX..

[6] Steinmetz Z., Kintzi A., Muñoz K., Schaumann G.E. (2020). A simple method for the selective quantification of polyethylene, polypropylene, and polystyrene plastic debris in soil by pyrolysis-gas chromatography/mass spectrometry. J. Anal. Appl. Pyrolysis..

[7] Primpke S., Fischer M., Lorenz C., Gerdts G., Scholz-Böttcher B.M. (2020). Comparison of pyrolysis gas chromatography/mass spectrometry and hyperspectral FTIR imaging spectroscopy for the analysis of microplastics. Anal. Bioanal. Chem..

[8] Frontier Laboratories (2021). PYA1-147E Polymer Library for Pyrolysis-GC/MS (Version 1).

[9] Frontier Laboratories (2021). PYA1-148E Additives and Polymer-Related Compounds Library for Pyrolysis-GC/MS (Version 1).

[10] Liu M., Song Y., Lu S., Qiu R., Hu J., Li X., Bigalke M., Shi H., He D. (2019). A method for extracting soil microplastics through circulation of sodium bromide solutions. Sci. Total. Environ..

[11] Bartnick R., Rodionov A., Oster S.D.J., Löder M.G.J., Lehndorff E. (2024). Plastic quantification and polyethylene overestimation in agricultural soil using large-volume pyrolysis and TD-GC–MS/MS. Env. Sci. Technol..

