# Supplementary material for: A practical method for mass quantification of microplastics in soil media using pyrolysis gas chromatography-mass spectrometry
Source: MethodsX. 2025 Nov 5;15:103711. doi: 10.1016/j.mex.2025.103711 (PMC12664816; doi:10.1016/j.mex.2025.103711)
Supplement: Supplementary file 1 [file mmc1.docx]

# **Supplementary Materials:**

**Tracing the Invisible: A practical comprehensive method for Mass Quantification of Microplastics in soil samples using Py-GC/MS and FTIR"**

Elham Faraji^1^, Patricia Cabedo-Sanz, Ajit K Sarmah

Department of Civil and Environmental Engineering, The Faculty of Engineering, The University of Auckland, New Zealand

**^1^Corresponding Author’s Address:**

Department of Civil and Environmental Engineering,

The Faculty of Engineering, The University of Auckland, New Zealand.

Email: Elham.faraji@auckland.ac.nz

**Table SM 1**- Microplastics Calibration Standard (MPs-CaCO3)

| Polymer / Diluent | | Quantitative amount in MPs-CaCO_3_ (4mg) | | Chemical structure |
| --- | --- | --- | --- | --- |
| Name | Abbr. |  |  |  |
| Polyethylene | PE | 163.4 | µg | 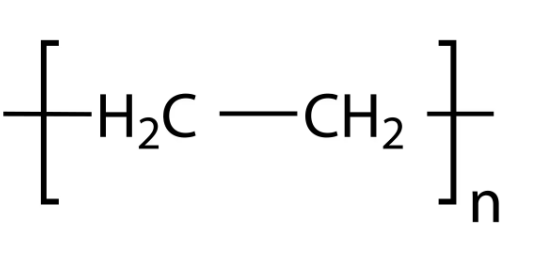   \|  \| \| --- \| |
| Polypropylene | PP | 39.7 | µg | \| 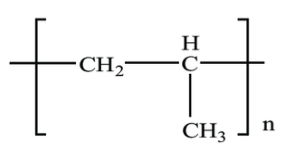 \| \| --- \| |
| Polystyrene | PS | 9.2 | µg | 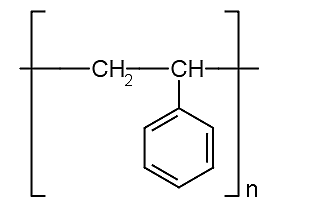   \|  \| \| --- \| |
| Acrylonitrile butadiene styrene Polymer | ABS | 14 | µg | 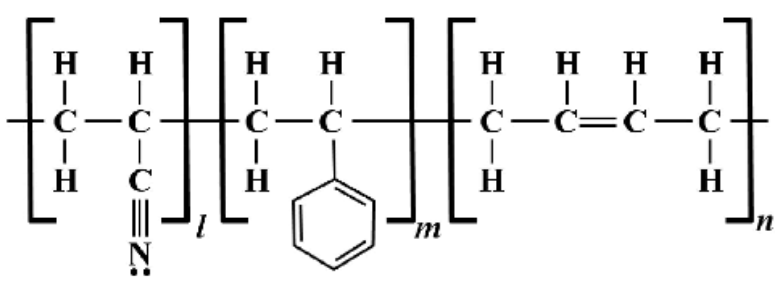   \|  \| \| --- \| |
| Styrene-butadiene rubber | SBR | 18.5 | µg | 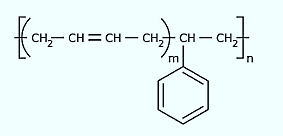   \|  \| \| --- \| |
| Polymethyl methacrylate | PMMA | 7.9 | µg | 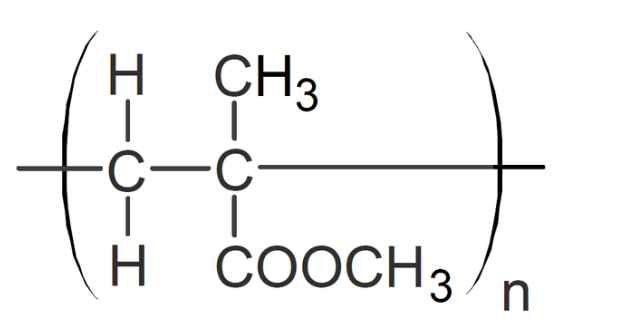   \|  \| \| --- \| |
| Polycarbonate | PC | 4.9 | µg | 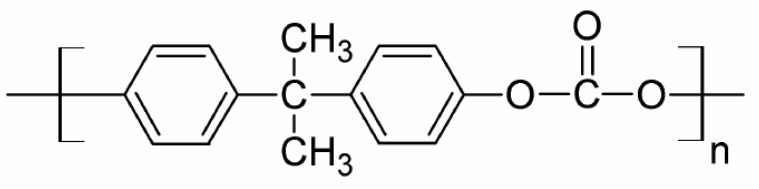   \|  \| \| --- \| |
| Polyvinylchloride | PVC | 39.4 | µg | 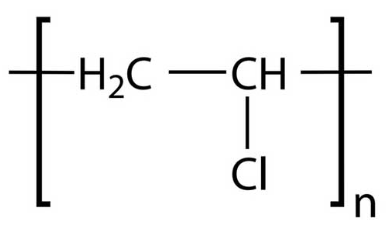   \|  \| \| --- \| |
| Polyurethane | PU | 1.8 | µg | 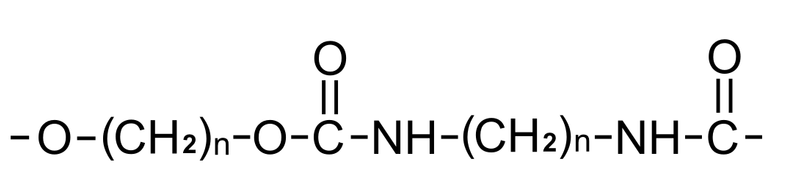   \|  \| \| --- \| |
| Polyethylene terephthalate | PET | 17.5 | µg | 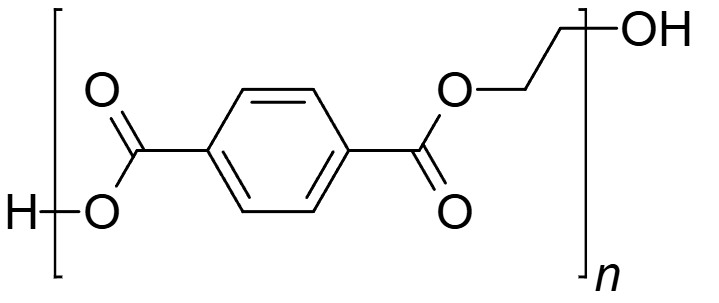   \|  \| \| --- \| |
| Nylon-6 | N-6 | 4.5 | µg | 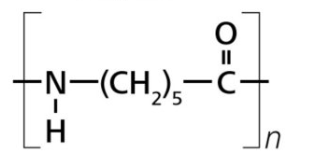   \|  \| \| --- \| |
| Nylon-6,6 | N-66 | 18.9 | µg | \| 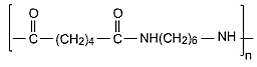 \| \| --- \| |
| Calcium carbonate | CaCO_3_ | - | | - |

**Table SM2**: Characteristics of the Polymers using as spiked MPs from Avient, New Zealand.

| Material | Source/Usage | Condition | Density (g/cm^3^) |  |
| --- | --- | --- | --- | --- |
| HPP powder | Standard Polymers from Avient | Fresh | 0.9 |  |
| HIPS powder | Standard Polymers from Avient | Fresh | 1.04 |  |
| LLDPE powder | Standard Polymers from Avient | Fresh | 0.936 |  |

**Table SM3**: FTIR operating conditions

| Accessory | A225/Q Platinum ATR, Multiple Crystals CRY:Diamo | | |
| --- | --- | --- | --- |
| Resolution | 4 cm^-1^ | **External synchronisation** | off |
| Sample scan time | 16 scans | **Source settling** | MIR |
| Background scan time | 16 scans | **Beam splitter** | Wide Range T MIR-FIR |
| Result spectrum | Absorbance | **Optical Filter setting** | open |
| Wave data | 600-4500 cm^-1^ | **Aperture setting** | 6mm |
| Measurement channel | Sample Compartment | **Scanner velocity** | 10 KHz |
| Detector setting | RT-DLa TGS Wide Range (Internal pos.1) | | |

**Table SM 4**: GCMS operating conditions

| Gas Chromatography | GC-2030 |
| --- | --- |
| Injection port mode | Split mode, 50:1 split ratio |
| Carrier gas | Helium |
| Injection port temperature (^o^C) | 300 |
| Column | Ultra Alloy Capillary Col., 30 m x 0.25 mmID x 0.25 μm |
| Flow control mode | Pressure (65.0 kPa) |
| Oven Temperature | 40 °C (2.0 mins.), 20 °C /mins. to 320 °C (10mins) |
| Mass Spectrometer | **GCMS-QP2010S Shimadzu** |
| Interface Temperature (^o^C) | 320 |
| Ion Source Temperature (^o^C) | 230 |
| Detector Voltage (kV) | Relative to Tune -0.1 |
| Threshold | 100 |
| Scan Range | m/z 29 to 400 Scan Speed 2000 |
| Ionization energy | 70 ev |
| Pyrolyzer | **EGA/PY-3030D Frontier Labs** |
| Single Shot Furnace Temp (^o^C) | 600 |
| Interface Temp (^o^C) | 300 |
| Pyrolysis holding time | 0.2 min |
| Selective Sampler gas | Helium |
| Flow rate of sampler gas | 1.04 mL/min |
| Backflush system |  |
| Analysis mode pressure (0 – 20 min) | Inlet pressure: 65.0 kPa  APC (Backflush pressure): 5.0 kPa |
| Backflush mode pressure (20 – 26 min) | Inlet pressure: 20.0 kPa  APC (Backflush pressure): 120.0 kPa |

**Figure SM1**: Example of a total ion chromatogram (TIC) for Standard Microplastics-CaCO_3_ Frontier Lab Calibration Kit using Py-GC/MS.

**Table SM5**- Characteristic pyrolyzate compounds of the target plastic polymers, respective indicator ions and retention time under the defined PY-GC/MS conditions. Pyrolyzate compounds marked by “*” were used for quantification

| **Polymer** | **Pyrolyzate Compounds** | **Indicator Ion (m/z)** | **Tr (min)** |
| --- | --- | --- | --- |
| PE | 2,4,6,8-Tetramethyl-1-undecene | 97 | 12.11 |
|  | n-Hexadec-1-ene (C16:1) | 83 | 10.52 |
|  | 1-Dodecene (C12:1) | 83 | 8.94 |
|  | **1,20-Heneicosadine *** | **82** | **16.217** |
| PP | **2,4-Dimethyl-1-heptene *** | **126** | **6.597** |
|  | 2,4,6-Trimethyl-1-nonene | 97 | 9.73 |
| PS | Styrene | 104 | 7.37 |
|  | Styrene dimer: 3-butene-1,3-diyldibenzene | 130 | 13.75 |
|  | **Styrene trimer: 5-hexene-1,3,5-triyltribenzene *** | **91** | **14.83** |
| ABS | 4-Methylstyrene | 118 | 10.56 |
|  | **2-Phenethyl-4-phenylpent-4-enenitrile *** | **170** | **17.683** |
| SBR | Butadiene | 54 | 6.28 |
|  | 4-Methylcyclohexene | 117 | 7.77 |
|  | **4-Phenylcyclohexene *** | **104** | **7.34** |
| PMMA | **Methyl methacrylate *** | **100** | **4.73** |
| PC | **P-Isoprpenylphenol *** | **134** | **11.477** |
|  | p-Cresol | 107 | 6.77 |
|  | p-Ethylphenol | 107 | 7.94 |
| PVC | Toluene | 91 | 5.1 |
|  | Benzene | 78 | 5 |
|  | **Naphtalene *** | **128** | **10.64** |
| PU | p-Isopropenylphenol | 134 | 8.17 |
|  | **4,4-Methylenedianiline *** | **198** | **17.395** |
| PET | Benzene | 78 | 5.42 |
|  | Benzoic acid | 122 | 10.08 |
|  | **Benzophene *** | **182** | **14.22** |
| N-6 | **ε-Caprolactam *** | **113** | **11.63** |
|  | 2-Cyclopenten-1-one | 114 | 11.36 |
| N-66 | **Cyclopentanone *** | **84** | **5.863** |


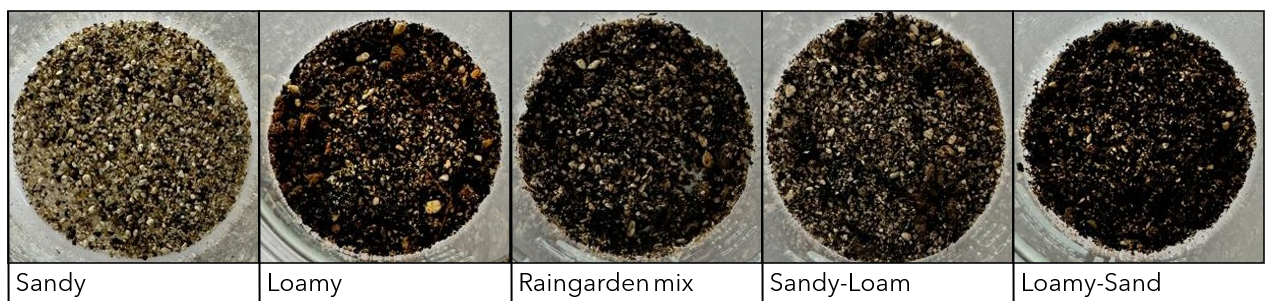


**Figure SM2**: Synthesized soil media-based media samples spiked by MPs.


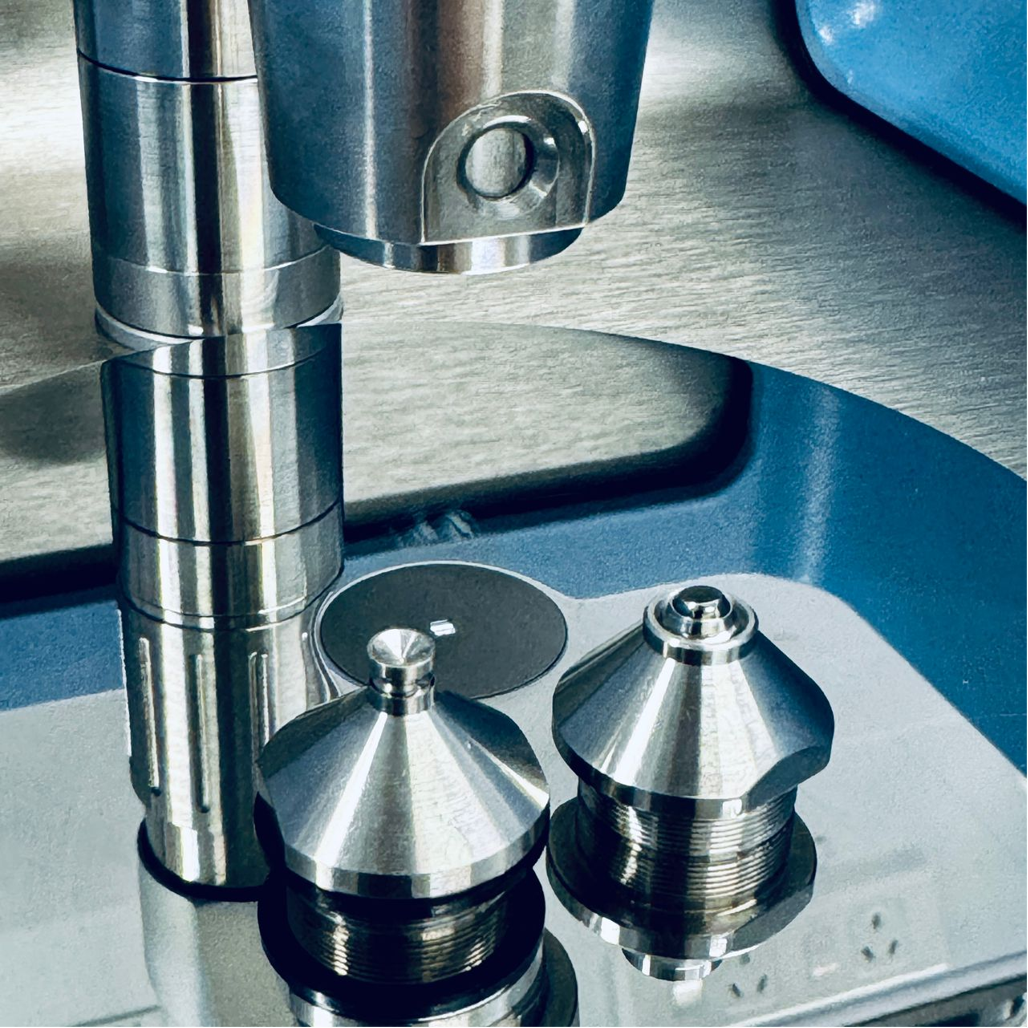


**Concave sample holder**

**Flat sample holder**

**Figure SM3**: Concave and flat sample holder parts of ATR-FTIR.

**PE**

**PP**


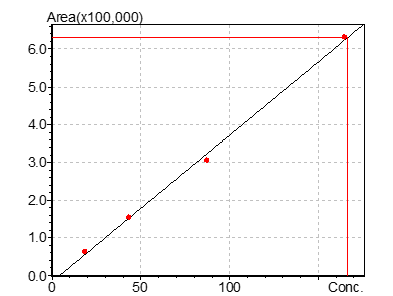

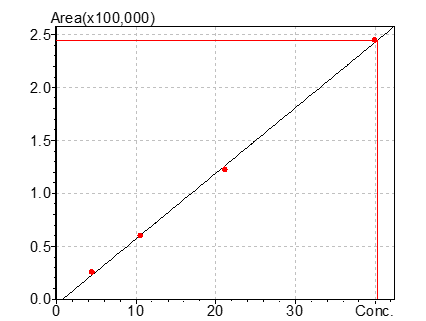

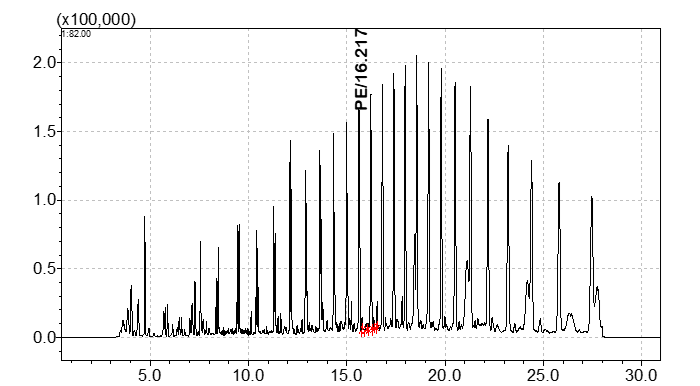

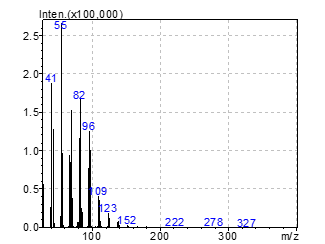

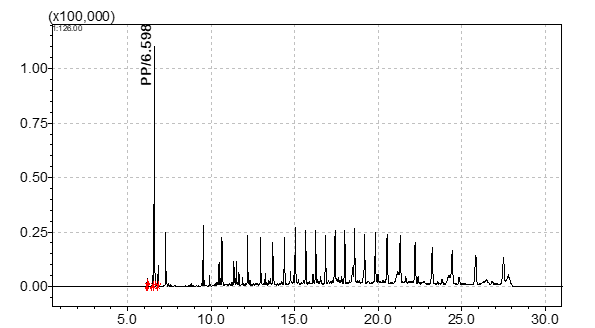

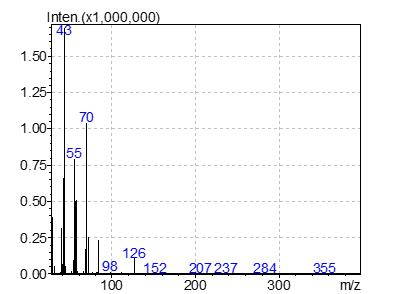

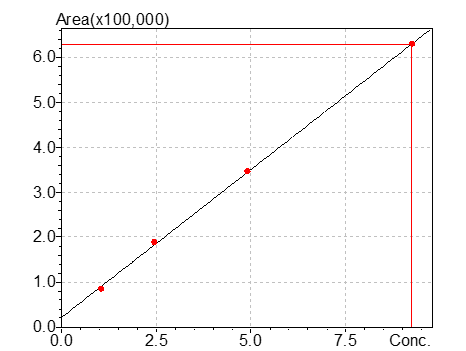

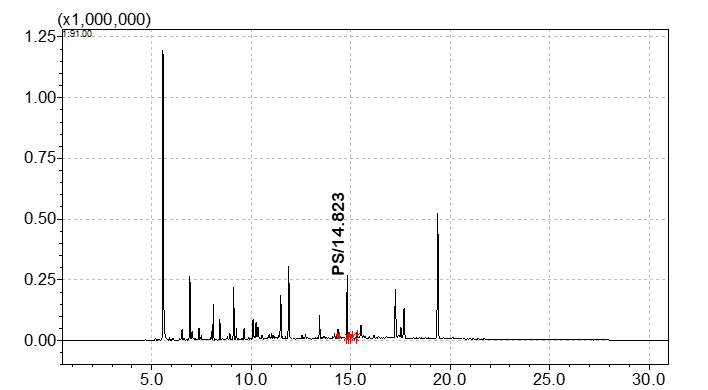

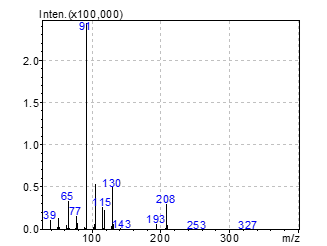

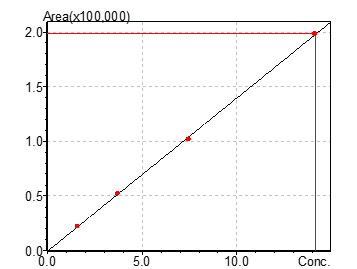

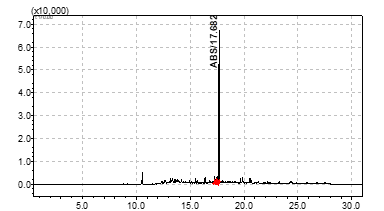

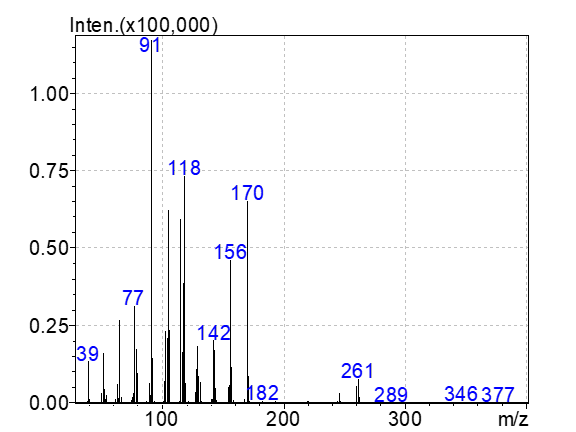

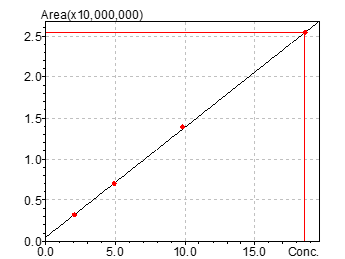

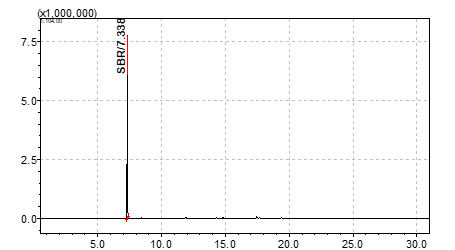

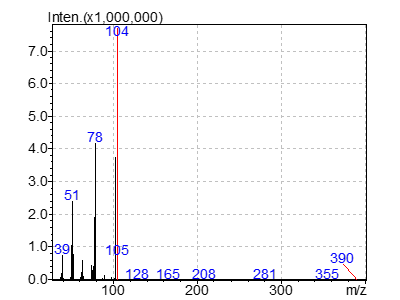

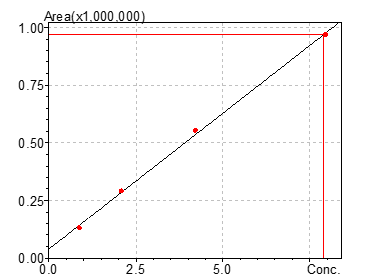

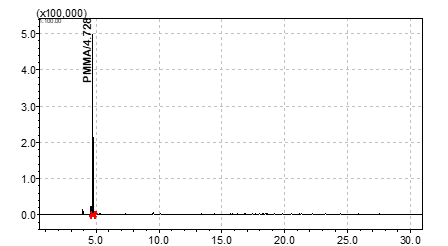

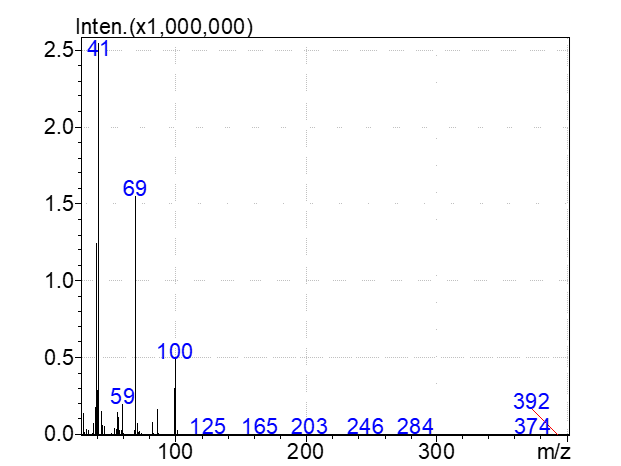

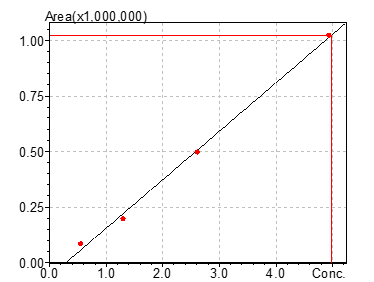

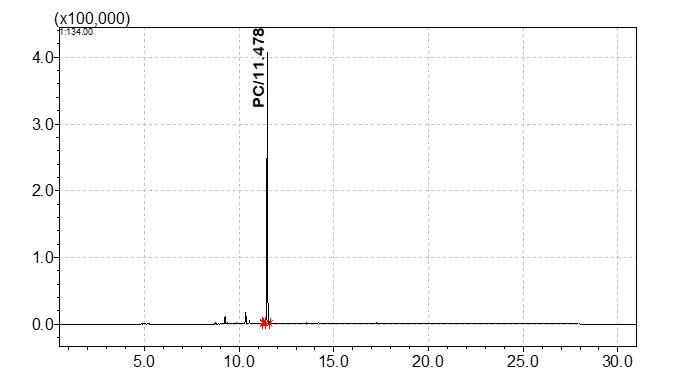

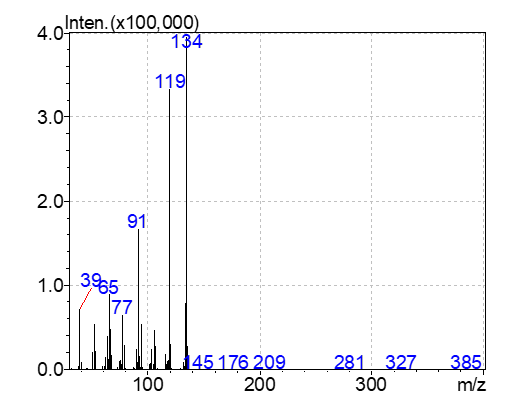

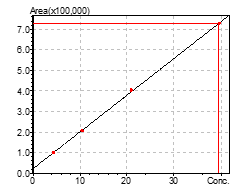

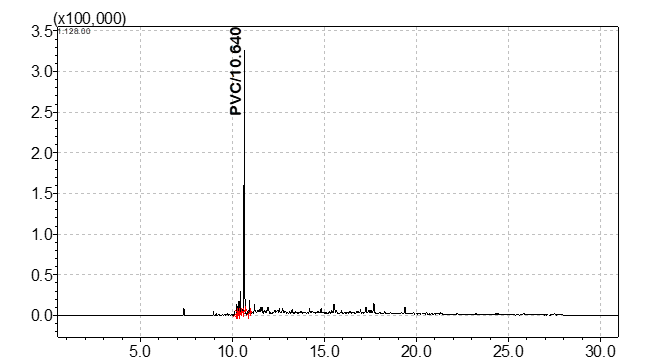

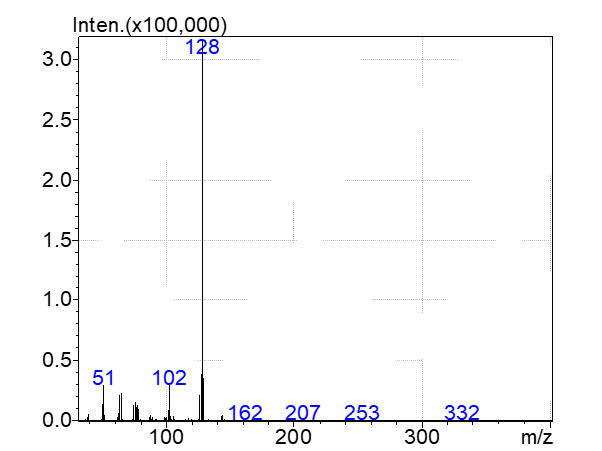

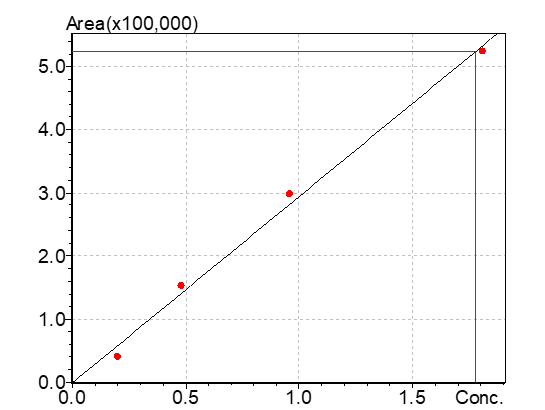

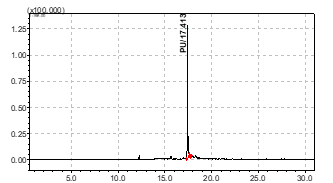

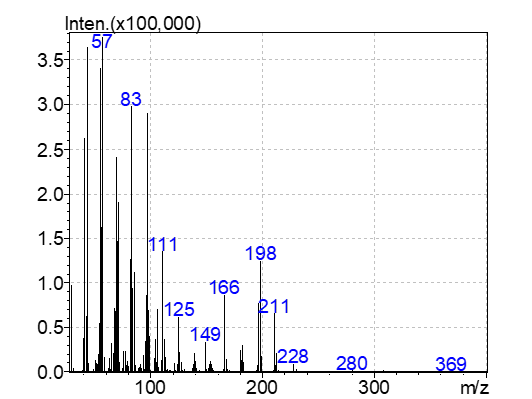

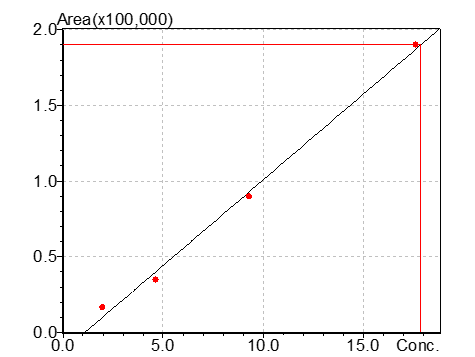

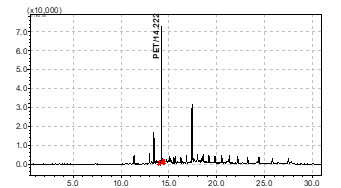

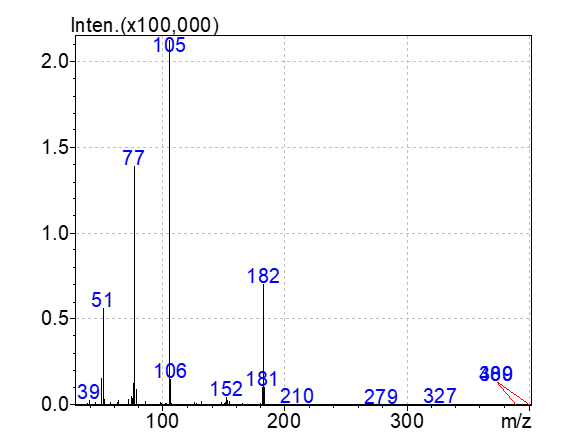

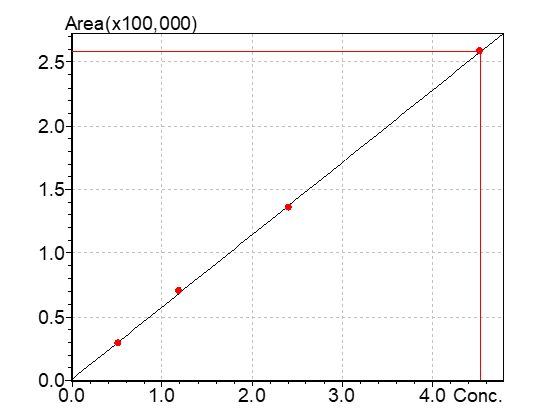

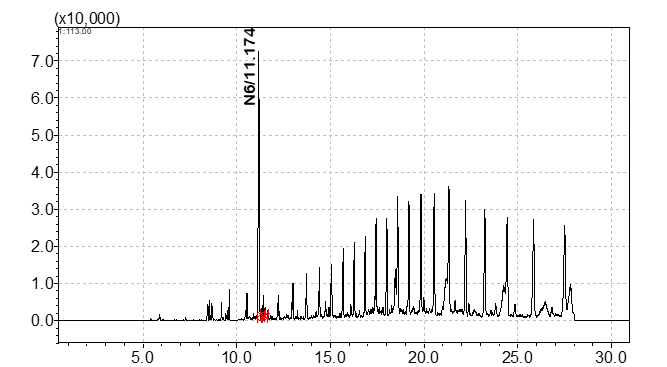

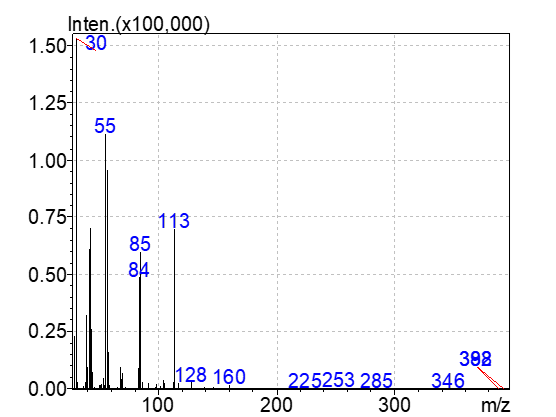

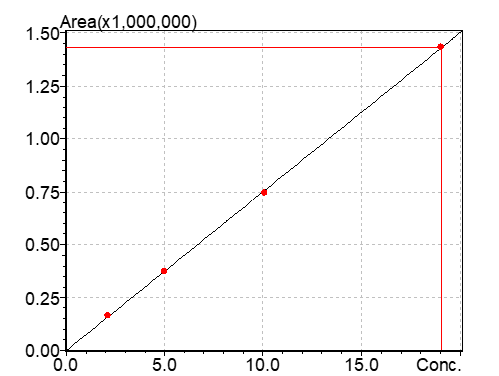

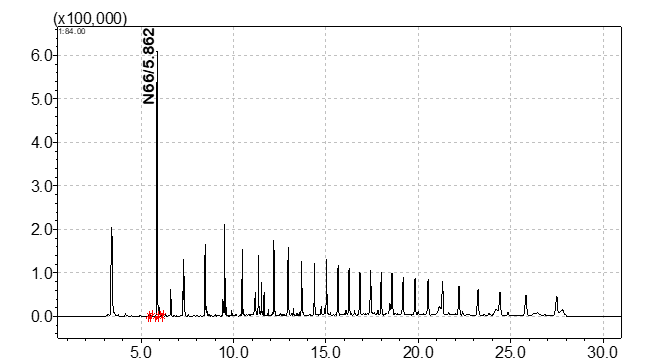

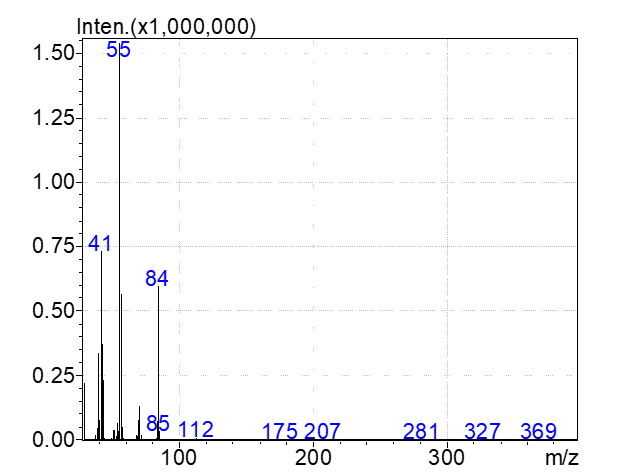


Y = (56783.3)X + (955.721)

R^2^=0.999

R^2^=0.9976

Y = (6187.07)X + (-4759.77)

Y = (3877.12)X + (-15168.8)

**ABS**

**PS**

R^2^=0.9995

R^2^=0.9995

Y = (13995.4)X + (-440.547)

Y = (65767.1)X + (22425.1)

**PMMA**

**SBR**

R^2^=0.9985

R^2^=0.9998

Y = (1.33866e+006)X + (505798)

Y = (117789)X + (39318.5)

**PC**

**PVC**

R^2^=0.9996

R^2^=0.9973

Y = (217569)X + (-61797.4)

Y = (17869.8)X + (22300.8)

**PET**

**PU**

Y = (11324.0)X + (-12136.4)

Y = (295197)X + (-1150.24)

R^2^=0.9948

R^2^=0.9939

**N6**

**N66**

R^2^=0.9998

R^2^=0.9997

Y = (75132.8)X + (-487.504)

**Figure SM4**: Calibration Curves for 12 polymers created using Standard Microplastics-CaCO_3_ Frontier Lab Kit.


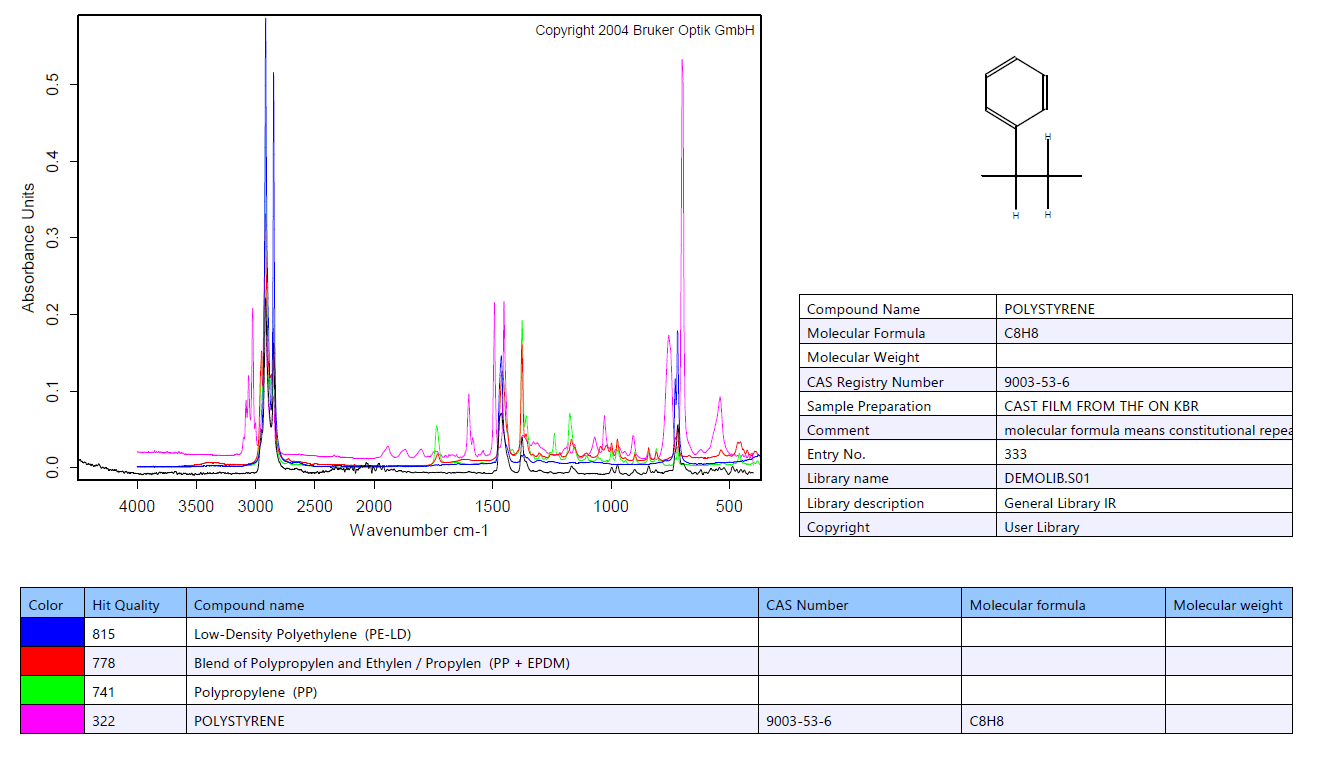


**Figure SM5:** Result of OPUS8.5.29 libraries for identification of LLDPE, HPP and HIPS extracted from synthetic soil media sample.


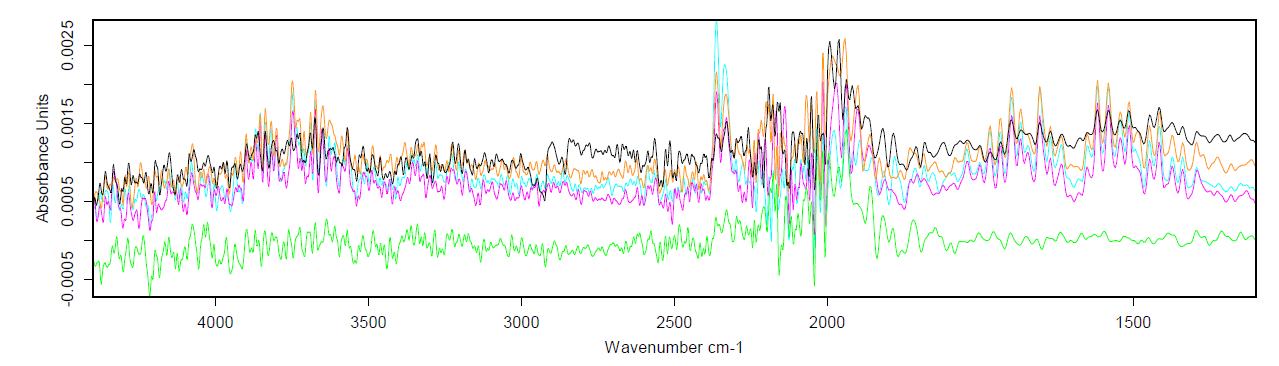


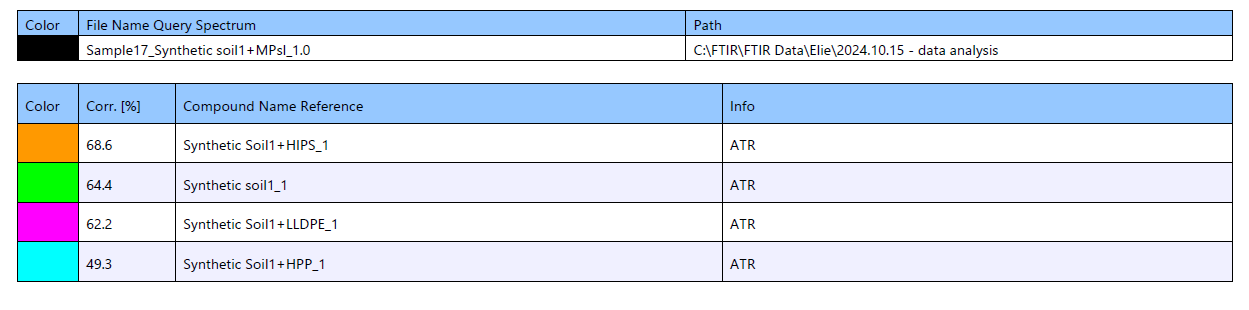


**Figure SM6:** Quick library search (OPUS 8.5.0 software) for MPs identification in synthesized soil media samples after FTIR analysis.


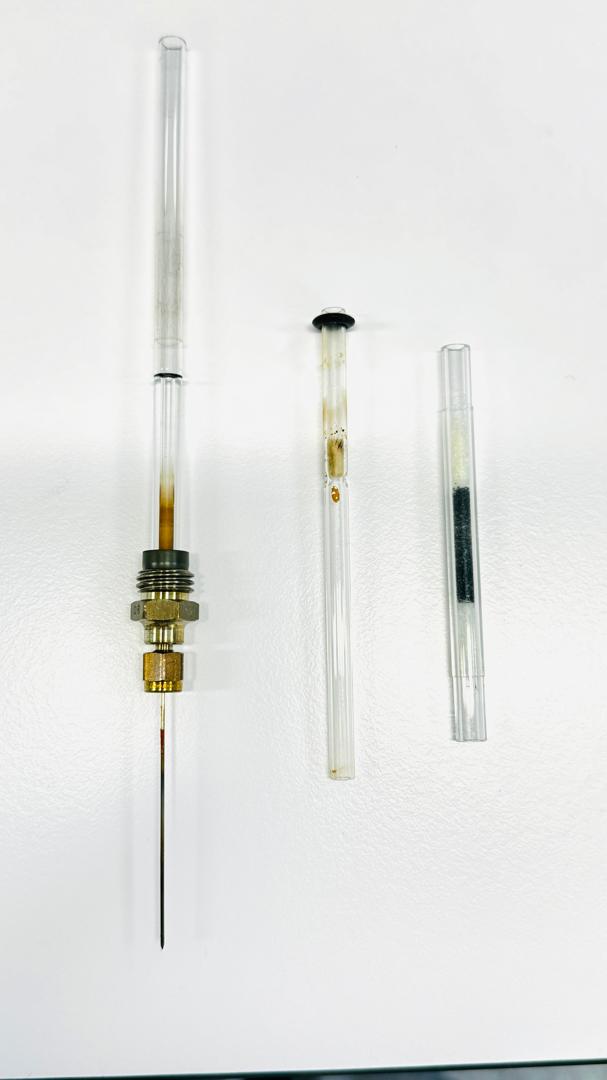


**Figure SM7:** Contaminated parts of the pyrolyser unit and gas chromatograph.

**SM Polymer Approval:**

Data file analysis was imported from the Postrun software into the F-Search software and peaks and mass spectra were detected to view chromatographic peaks separately by inputting the indicator ions for each polymer. Table SM4 presents the full chromatogram and the indicator ions for PP identification, including 126 m/z and 97 m/z. By clicking on 6.597 min for ion 126 m/z and 9.73 min for ion 97 m/z, search the library for 2,4-Dimethyl-1-heptene and 2,4,6-Trimethyl-1-nonene (as shown in Tables SM5 and SM6). If these pyrolyzates are identified, polymer identification is confirmed.


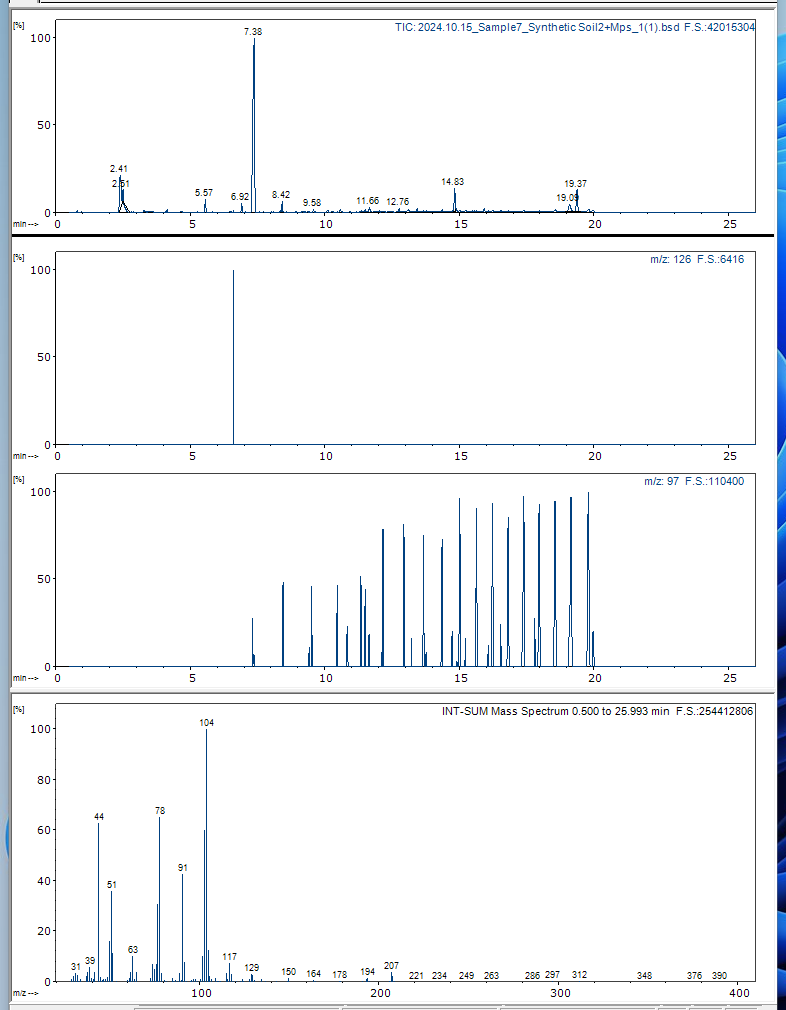


**Figure SM8**- Py-GC/MS analysis data for Loamy synthetic soil, identifying PP polymer.


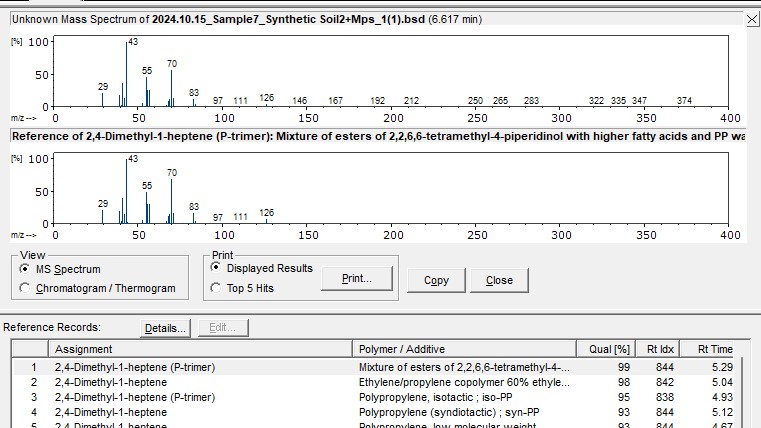


**Figure SM9**- Pyrolyzate identification (ion 126 m/z) using F-Search software for Loamy synthetic soil for PP identification.


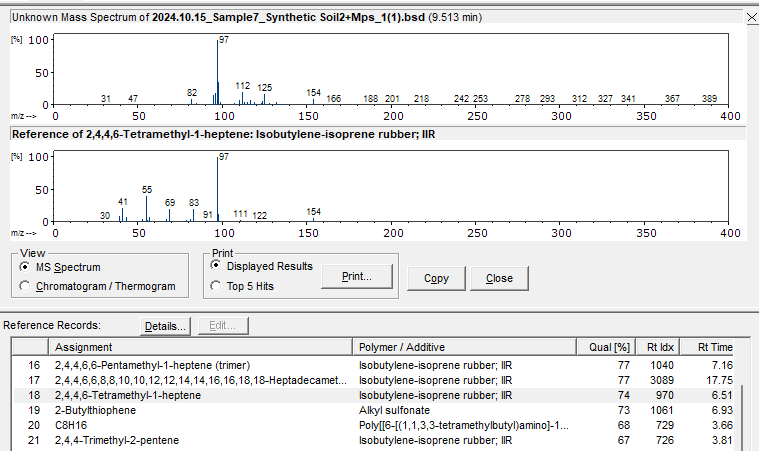


**Figure SM10-** Pyrolyzate identification (ion 97 m/z) using F-Search software for Loamy synthetic soil for PP identification.


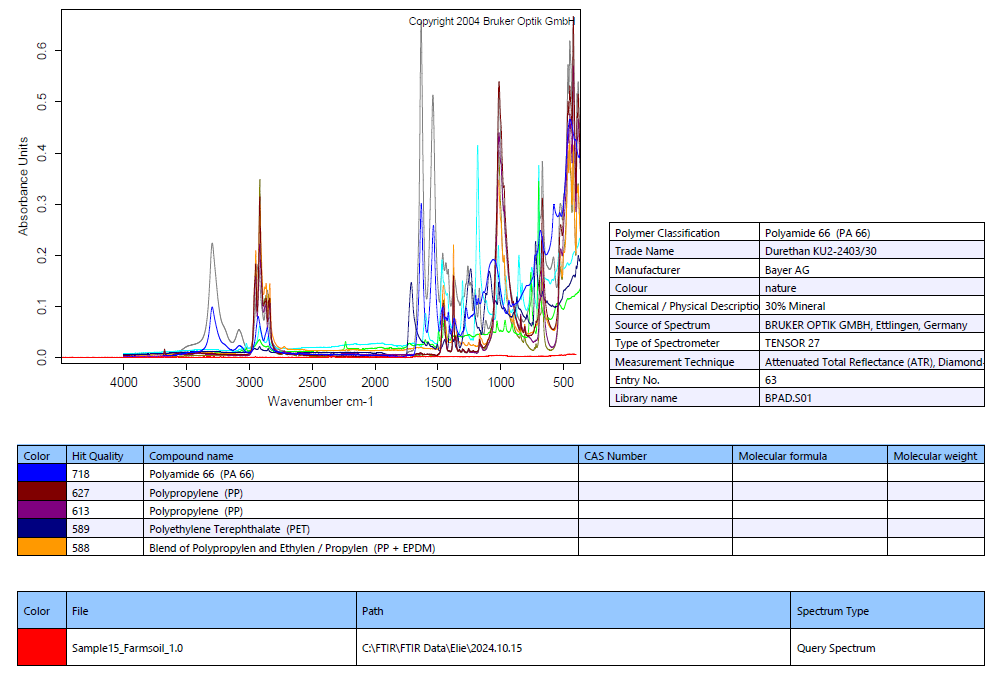


**Figure SM11-** Result of OPUS8.5.29 libraries for identification of Microplastics in environmental Farm soil sample.


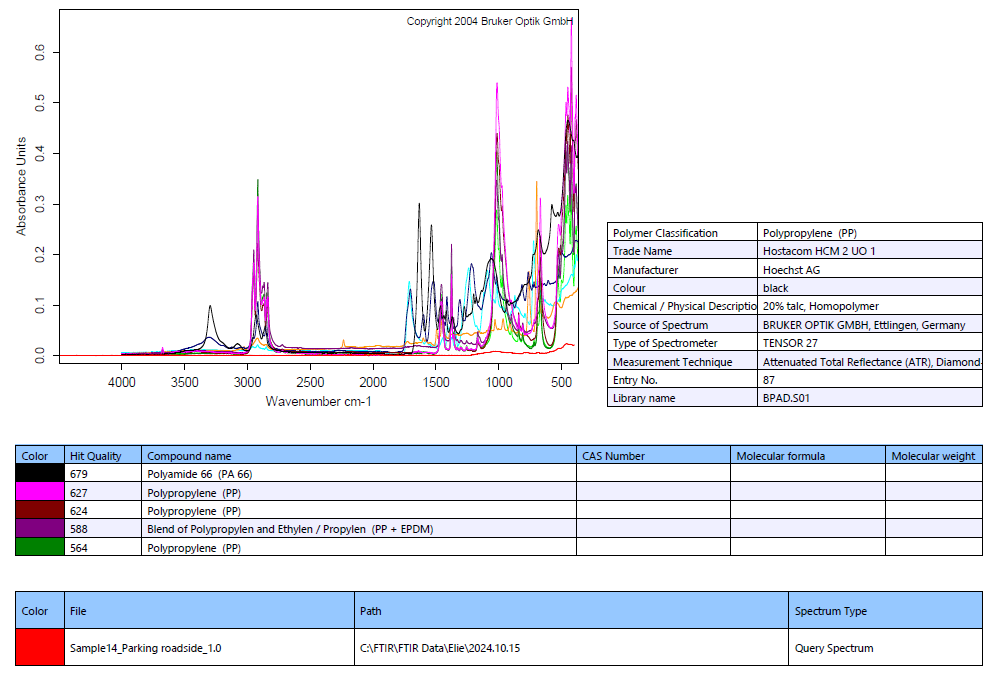


**Figure SM12-** Result of OPUS8.5.29 libraries for identification of Microplastics in environmental Parking roadside soil sample.

**Figure SM13-** Total ion chromatogram (TIC) for environmental Farm soil sample using Py-GC/MS.

**Figure SM14-** Total ion chromatogram (TIC) for environmental parking roadside sample using Py-GC/MS.
